# Mechanistic Insights into Piperine-Driven Oxidative Stress, Autophagy Activation and Anti-Migration Effects in Caco-2 Cells

**DOI:** 10.3390/molecules31071106

**Published:** 2026-03-27

**Authors:** Hla Sudan, Sofia Passaponti, Ilenia Casini, Roberta Romagnoli, Laura Cresti, Mariangela Gentile, Maria Frosini, Anna Maria Aloisi

**Affiliations:** 1 Department of Medicine, Surgery and Neuroscience, University of Siena, 53100 Siena, Italy; sofia.passaponti2@unisi.it (S.P.); ilenia.casini2@unisi.it (I.C.); 2 Department of Life Sciences, University of Siena, 53100 Siena, Italy; roberta.romagnoli@unisi.it (R.R.); laura.cresti@unisi.it (L.C.); mariangela.gentile@unisi.it (M.G.); maria.frosini@unisi.it (M.F.)

**Keywords:** piperine, autophagy, colon cancer, oxidative stress, migration

## Abstract

**Background**: Piperine, an alkaloid from *Piper nigrum*, modulates oxidative stress, proliferation, and survival pathways in several cancer models; however, its mechanistic effects in colorectal epithelial Caco-2 cells remain insufficiently defined. **Objective**: This study aimed to investigate the cytotoxic, antiproliferative, oxidative, autophagic, and anti-migratory effects of piperine in Caco-2 cells. **Methods**: Caco-2 cells were treated with piperine (0.001–0.1 mg/mL) for up to 72 h. Cell viability, proliferation, and migration were assessed using SRB and scratch assays. Oxidative stress, apoptosis, autophagy, and tight junction integrity were evaluated through ROS quantification, Western blotting, gene expression analysis, confocal microscopy, and transmission electron microscopy (TEM). NACET was used to determine the contribution of oxidative stress to piperine-induced cytotoxicity and autophagy. **Results**: Piperine induced a time- and dose-dependent reduction in viability, with viability decreasing to 53.0 ± 2.88% at 0.1 mg/mL after 72 h. Proliferation decreased to 51% of control levels (*p* < 0.001), accompanied by p21 upregulation (*p* < 0.05), indicating G2/M cell cycle arrest. Piperine markedly increased intracellular ROS (*p* < 0.001), downregulated NRF2 (*p* < 0.05), and suppressed *GSTA1* expression (*p* < 0.001), while NACET co-treatment restored viability (*p* < 0.001). No activation of caspase-dependent apoptosis was observed. Piperine significantly enhanced autophagic flux, as shown by the increased LC3B-II/LC3B-I ratio (*p* < 0.01), elevated LC3B-II/LAMP-1 co-localization (*p* < 0.01), and chloroquine-induced accumulation of LC3B-II and p62 (*p* < 0.01), with preserved lysosomal function. TEM analysis confirmed a marked increase in double-membrane autophagosomes in piperine-treated cells compared with controls. NACET reduced LC3B-II/LC3B-I levels, increased p21 expression, and significantly improved cell viability, indicating that piperine-induced autophagy is cytotoxic and driven by oxidative stress. Additionally, piperine upregulated occludin (*p* < 0.01) and reduced cell migration independently of proliferation (*p* < 0.01). **Conclusions**: Piperine exerts antiproliferative effects in Caco-2 cells through ROS-mediated stress, p21-dependent G2/M arrest, and activation of cytotoxic autophagy. Its ability to impair migration and enhance tight junction integrity further supports its potential as a complementary therapeutic agent in colon cancer.

## 1. Introduction

Colorectal cancer ranks as the third most prevalent cancer globally, representing around 10% of all cancer diagnoses. It is also the second leading cause of cancer-related mortality worldwide, according to the World Health Organization (2026) [1]. In the United States, the American Cancer Society predicts approximately 108,860 new cases of colon cancer in 2026 [2]. In the late 1990s, colorectal cancer ranked as the fourth leading cause of cancer-related mortality among men and women under the age of 50; however, it has since become the leading cause in men and the second leading cause in women [3]. This cancer can originate in the colon—the largest segment of the large intestine—or in the rectum.

Chemotherapeutic agents have shown enduring clinical efficacy over decades in the management of colon cancer, especially in early stages [4]. However, chemotherapy resistance remains a significant challenge, limiting treatment efficacy and contributing to high mortality rates [5].

In this context, natural compounds have emerged as promising candidates due to their ability to modulate multiple cellular processes necessary for cancer cell growth [6].

Piperine (1-piperoyl piperidine), an amide alkaloid extracted from black pepper (*Piper nigrum*) and long pepper (*Piper longum*), has demonstrated significant cytotoxic and cytostatic effects in various cancer cell lines [7]. It inhibits tumor growth by targeting multiple pathways, including inducing cell cycle arrest and apoptosis in breast cancer [8], overcoming doxorubicin resistance via the PI3K/Akt/mTOR pathway in triple-negative breast cancer [9], and inducing apoptosis and autophagy in oral squamous carcinoma cells by inhibiting PI3K/protein kinase activity [10]. In colon cancer, piperine suppresses growth of the HT-29 cells via G1 arrest and apoptosis mediated by endoplasmic reticulum stress [11].

Reactive oxygen species (ROS) are increasingly recognized as key regulators of cancer development, with effects that can either support or suppress tumor growth depending on their abundance. At modest concentrations, ROS act as secondary messengers that enhance malignant behaviors, including increased proliferation, enhanced motility, and reduced sensitivity to therapy. When ROS accumulate beyond the cell’s buffering capacity, they generate oxidative stress that activates stress response pathways and damages essential biomolecules and can drive cells toward death [12]. This stress triggers the DNA damage response, pushing cancer cells into cell cycle arrest [13]. To counteract oxidative stress, cells activate adaptive antioxidant programs, among which nuclear factor erythroid 2-related factor 2 (NRF2) plays a central role by regulating redox homeostasis and antioxidant gene expression [14].

Autophagy represents a fundamental adaptive response that enables cells to cope with various forms of stress. In the context of cancer, this process has a multifaceted role, functioning either to sustain tumor cell survival or to contribute to cell death depending on the cellular environment [15,16]. By degrading and recycling intracellular components, autophagy helps maintain metabolic balance during unfavorable conditions. However, when activated beyond a certain threshold, this pathway can shift from a protective mechanism to one that promotes cell demise, offering a potential therapeutic avenue for targeting tumors that are resistant to apoptosis.

Although piperine has been reported to induce ROS generation, cell cycle arrest, and autophagy in several cancer models, including colon cancer, its effects in Caco-2 cells have not been characterized. Moreover, no studies have examined whether piperine enhances autophagic flux beyond starvation-induced levels, nor whether its modulation of occludin expression contributes to altered migratory behavior. Addressing this question is particularly important because Caco-2 cells carry characteristic mutations in key oncogenes and tumor suppressors, including APC, CTNNB1, and KRAS, which drive colorectal tumorigenesis and may alter therapeutic responses compared with other colon cancer models [17,18,19,20,21]. Consequently, examining how piperine influences oxidative stress and autophagy in this genetic context is essential for advancing our understanding of its therapeutic potential and mode of action in colorectal cancer.

## 2. Results

### 2.1. Experimental Results

#### 2.1.1. Piperine Exhibits Dose- and Time-Dependent Cytotoxic and Antiproliferative Effects in Caco-2 Cells

**Figure 1:**
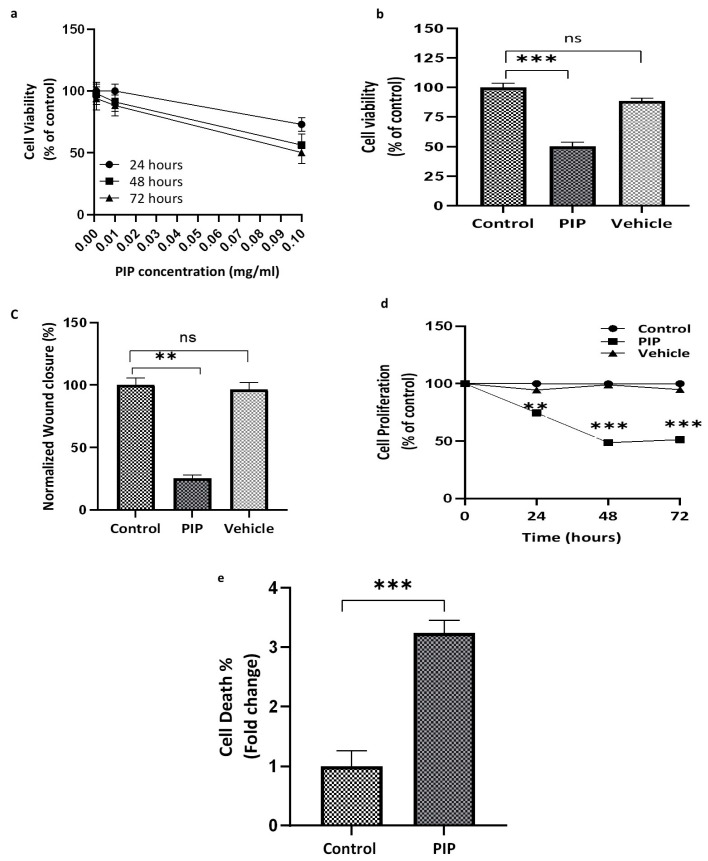
**Piperine reduces the viability and proliferation of Caco-2 cells.** (**a**) SRB assay results show a time- and dose-dependent decrease in cell viability after piperine treatment. At 0.1 mg/mL for 72 h, viability dropped approximately to IC_50_. (**b**) At 0.1 mg/mL for 72 h, piperine reduced viability to 53.0 ± 2.88% vs. control (*p* < 0.001), while ethanol vehicle had no significant effect (98.0 ± 3.7%, *p* > 0.05). (**c**) The scratch assay showed significantly impaired in normalized wound closure in piperine-treated cells (25.6 ± 2.4%) compared to control (100 ± 4.68%) after 72 h of treatment (*p* < 0.01). (**d**) The SRB assay indicates significant inhibition of proliferation over time, with cell density decreasing to 51% of control at 72 h (*p* < 0.001). (**e**) Trypan blue exclusion analysis demonstrated a significant increase in non-viable cells following piperine treatment (*p* < 0.001). All values represent mean ± SEM (N = 3). Statistical significance: ns: not significant, ** *p* < 0.01 and *** *p* < 0.001 compared to control.

The cytotoxic effects of piperine on Caco-2 cells were first assessed using the SRB assay across a range of concentrations and incubation periods. Cells were treated with 0.001, 0.01, and 0.1 mg/mL piperine for 24, 48, and 72 h, respectively (Figure 1a). Piperine induced a clear dose- and time-dependent reduction in cell viability, with viability decreasing to 53.0 ± 2.88% at 0.1 mg/mL after 72 h (*p* < 0.001; 95% CI: 47.3% to 58.7%) (Figure 1a,b). Based on these results, 0.1 mg/mL was selected for subsequent experiments. The vehicle-treated group exhibited minimal cytotoxicity (2.0 ± 3.7%) relative to the untreated control, with no statistically significant difference (*p* > 0.05), confirming that the observed cytotoxicity was attributable to piperine rather than the vehicle.

To account for the contribution of proliferation to wound closure in the absence of a mitotic inhibitor, scratch assay values were normalized to the corresponding SRB signal obtained under identical treatment conditions. After 72 h of exposure to 0.1 mg/mL piperine, normalized wound closure was markedly reduced (25.60 ± 2.47%) compared with the control group (100 ± 4.68%) (*p* < 0.01) (Figure 1c). No significant difference was observed between the vehicle and control groups (*p* > 0.05).

As the assay was conducted without an antiproliferative agent, the reduced wound closure likely reflects a combined effect of impaired proliferation and diminished migratory capacity rather than a migration-specific response.

To further substantiate the antiproliferative activity of piperine, an additional SRB assay was performed in which absorbance was measured at 0, 24, 48, and 72 h. Piperine treatment resulted in significant time-dependent suppression of cell proliferation, reaching approximately 51% of control levels after 72 h (*p* < 0.001; 95% CI: 13.11% to 38.63%) (Figure 1d). In contrast, the vehicle group showed only a minimal, non-significant reduction relative to the control (*p* > 0.05).

Consistent with these findings, Trypan blue exclusion analysis demonstrated a significant increase in non-viable cells following piperine treatment, with approximately a three-fold elevation in cell death compared with the control group (*p* < 0.001; 95% CI: 1.56 to 2.92) (Figure 1e). These results indicate that the reduction in SRB signal reflects both a genuine cytotoxic effect and the inhibition of cell proliferation.

#### 2.1.2. Piperine Induces G2/M Cell Cycle Arrest in Caco-2 Cells Without Activating Apoptosis

**Figure 2:**
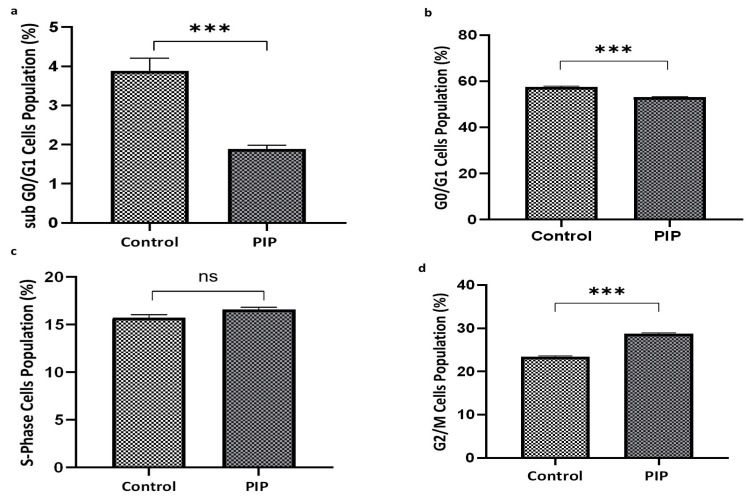
**Piperine induces G2/M cell cycle arrest in Caco-2 cells without triggering apoptosis.** Flow cytometry analysis of DNA content after 72 h of treatment. Piperine reduced the Sub-G0/G1 fraction (1.89 ± 0.09% vs. 3.88 ± 0.33%; *p* < 0.01) (**a**) and decreased G0/G1 phase cells (53.11 ± 0.14% vs. 57.46 ± 0.37%; *p* < 0.001) (**b**), while the S-phase remained unchanged (*p* > 0.05) (**c**). Significant accumulation of G2/M was observed (28.76 ± 0.23% vs. 23.34 ± 0.22%; *p* < 0.001) (**d**). Data represent mean ± SEM of three independent experiments. Statistical significance: ns: not significant and *** *p* < 0.001 compared to control.

Flow cytometric assessment of DNA content demonstrated clear alterations in cell cycle distribution between control and piperine-treated Caco-2 cells as presented in Figure 2. Piperine exposure significantly reduced the Sub-G0/G1 population (1.89 ± 0.09%) relative to the control group (3.88 ± 0.33%) (*p* < 0.01; 95% CI: 1.14 to 2.84), indicating the absence of apoptotic DNA fragmentation. A significant decrease in the proportion of cells in the G0/G1 phase was also observed following treatment (53.11 ± 0.14%) compared with controls (57.46 ± 0.37%) (*p* < 0.001; 95% CI: 4.32 to 6.97), whereas the S-phase fraction remained unchanged (*p* > 0.05; 95% CI: −1.83 to 0.09). In contrast, piperine induced a marked accumulation of cells in the G2/M phase (28.76 ± 0.23%) relative to the control group (23.34 ± 0.22%) (*p* < 0.001; 95% CI: −6.21 to −4.62).

**Figure 3:**
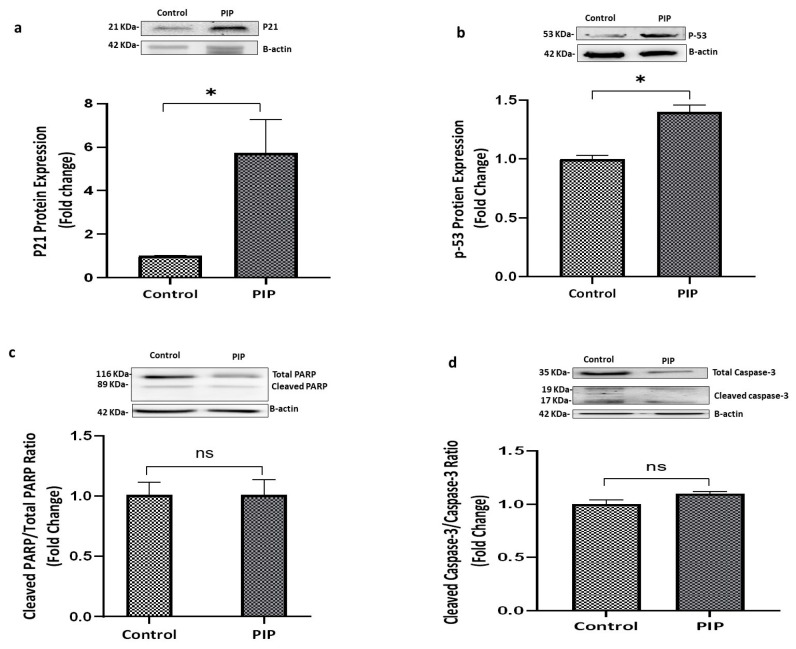
**Piperine Upregulates p21 and p53 Without Affecting PARP or Cleaved Caspase-3.** (**a**,**b**) Piperine upregulates the expression of p21 and p-53 proteins compared to control, indicating a cell cycle arrest (*p* < 0.05). (**c**,**d**) Cleaved/total PARP ratio and Cleaved Caspase-3/Caspase-3 ratio remained unchanged across groups (*p* > 0.05), suggesting apoptosis was not activated. All values represent mean ± SEM (N = 3). Statistical significance: ns: not significant and * *p* < 0.05 compared to control.

To further elucidate the molecular mechanisms underlying these cell cycle alterations, the expression of tumor protein p53 and the cyclin-dependent kinase inhibitor p21 (WAF1/Cip1) was examined. Piperine treatment resulted in a significant upregulation of both p53 (*p* < 0.05; 95% CI: −10.23 to −9.26) and p21 (*p* < 0.05; 95% CI: −11.27 to −1.57) compared with the control group, consistent with the induction of cell cycle arrest, as shown in Figure 3a,b.

The potential involvement of apoptosis was evaluated by measuring the expression of cleaved caspase-3, a key effector of both intrinsic and extrinsic apoptotic pathways, and the ratio of cleaved to total poly(ADP-ribose) polymerase (PARP), a canonical marker of programmed cell death. As illustrated in Figure 3d, cleaved caspase-3/caspase-3 ratio did not differ significantly between piperine-treated and control cells (*p* > 0.05). Similarly, the cleaved-PARP/total-PARP ratio remained unchanged (*p* > 0.05), as shown in Figure 3c. These findings indicate that piperine does not activate caspase-dependent apoptosis under the experimental conditions employed.

**Figure 4:**
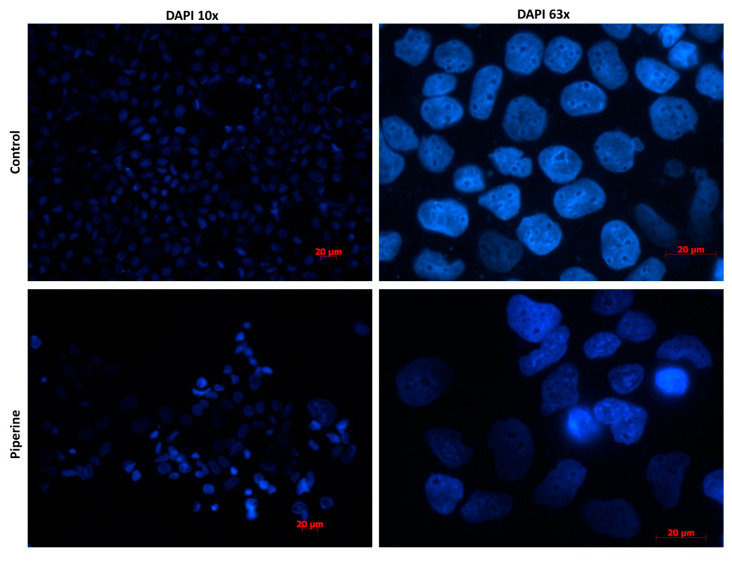
**Piperine does not induce nuclear morphological alterations in Caco-2 cells.** DAPI staining of control and piperine-treated Caco-2 cells imaged at 10× and 63× magnification. Nuclei appeared round, intact, and uniformly stained in both groups, with no evidence of chromatin condensation, nuclear fragmentation, or other apoptotic-associated abnormalities. Images are representative of three independent experiments.

Consistent with these results, DAPI staining revealed normal nuclear morphology in both control and piperine-treated cells (Figure 4). At 10× magnification, nuclei appeared uniformly distributed with comparable density across groups. Higher-resolution imaging at 63× confirmed these observations: nuclei were round, intact, and exhibited homogeneous DAPI fluorescence, with no evidence of chromatin condensation, nuclear fragmentation, or other morphological abnormalities. Collectively, these data indicate that piperine treatment does not induce detectable nuclear structural changes under the conditions tested.

#### 2.1.3. Piperine Activated Autophagy in Caco-2 Cells

**Figure 5:**
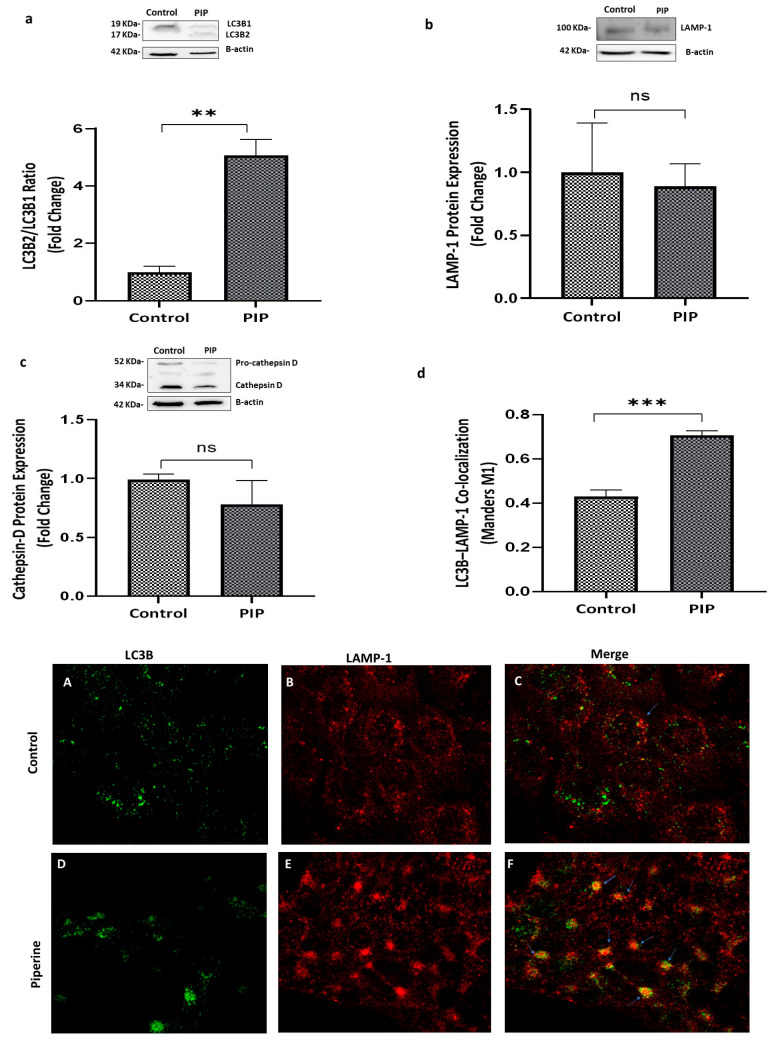
**Piperine Enhances Autophagosome–Lysosome Co-Localization Without Altering Lysosomal Protein Expression.** (**a**) Piperine treatment resulted in a significant increase in the LC3B-II/LC3B-I ratio compared with the control group (*p* < 0.01). (**b**) LAMP-1 protein levels did not differ significantly among the experimental groups (*p* > 0.05). (**c**) Cathepsin-D expression also remained unchanged, indicating that lysosomal function associated with apoptotic processing was preserved (*p* > 0.05). (**d**) Confocal microscopy demonstrated a marked increase in LC3B–LAMP-1 co-localization in piperine-treated cells (*p* < 0.001). Data are presented as mean ± SEM (N = 3). Statistical significance: ns: not significant, *** *p* < 0.001 and ** *p* < 0.01 vs. control. Confocal microscopy images showing LC3B (**A**), LAMP-1 (**B**), and LC3B–LAMP-1 co-localization (**C**) in control cells, and LC3B (**D**), LAMP-1 (**E**), and LC3B–LAMP-1 co-localization (**F**) in piperine-treated cells.

The conversion of the cytosolic form LC3B-I into the lipidated form LC3B-II was assessed. As illustrated in Figure 5a, this ratio was significantly increased in the piperine-treated group compared to the control group (*p* < 0.01; 95% CI: 2.47 to 5.41).

The observed increase in LC3B-II could result from either enhanced autophagic flux or a block in lysosomal degradation. To distinguish between these possibilities, the expression of LAMP-1 (a lysosomal marker) and cathepsin D (a lysosomal enzyme) was evaluated. No significant changes were observed in the piperine-treated group compared to the control group (*p* > 0.05), indicating that piperine does not affect lysosomal function as shown in Figure 5b,c.

**Figure 6:**
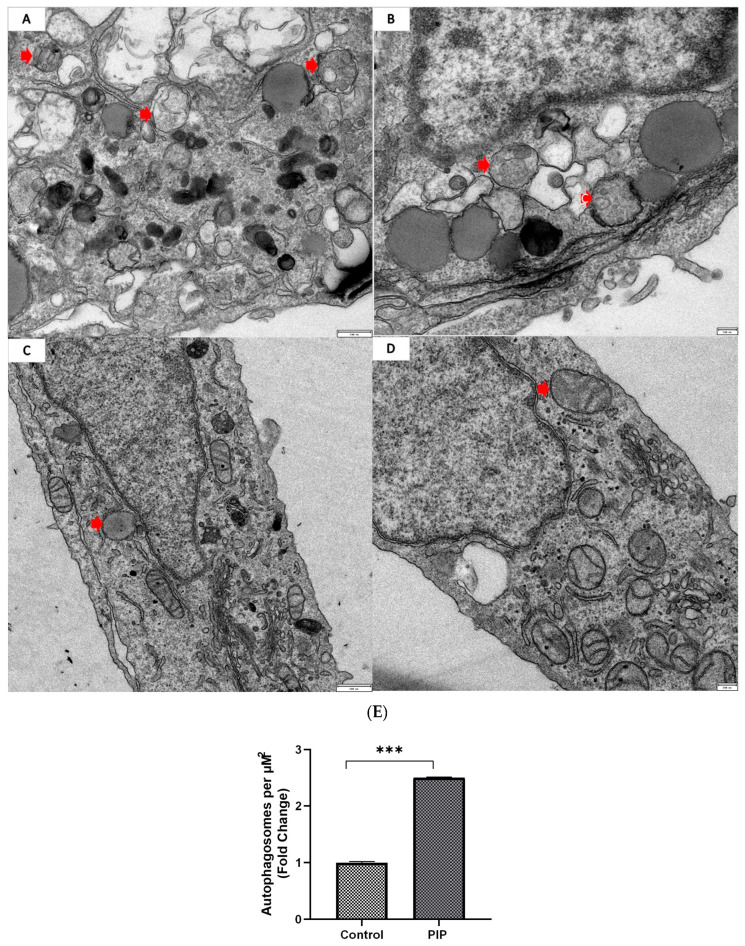
**Piperine increases autophagosome formation in Caco-2 cells.** Transmission electron microscopy images of control and piperine-treated Caco-2 cells. (**A**,**B**) Piperine treatment markedly increased the number of double-membrane autophagosomes (red arrows) compared with untreated controls (*p* < 0.001; 95% CI: 0.124–0.343). (**C**,**D**) Control cells displayed only occasional basal autophagic vacuoles, whereas piperine-treated cells exhibited abundant autophagic structures, confirming enhanced autophagosome formation at the ultrastructural level. Panels (**A**,**C**) include a 500-nm scale bar, while panels (**B**,**D**) include a 200-nm scale bar. (**E**) Autophagosomes formation increase significantly in piperine-treated cells (*p* < 0.001). Data are presented as mean ± SEM (N = 3). Statistical significance: *** *p* < 0.001 vs. control.

Additionally, confocal microscopy images confirmed a significant increase in autophagosome–lysosome fusion in the piperine-treated group, as indicated by enhanced LC3B-II–LAMP-1 co-localization (Mander’s M1, auto-thresholding = 0.708 ± 0.02) compared to the control group (Mander’s M1 = 0.430 ± 0.03) (*p* < 0.01, 95% CI: 0.186 to 0.370) (Figure 5d). Morever, TEM images revealed a clear increase in autophagic structures in piperine-treated cells compared with untreated controls. Treated cells displayed numerous double-membrane autophagosomes, consistent with active autophagic flux, whereas control cells showed only occasional basal autophagic vacuoles (*p* < 0.001, 95% CI: 0.124 to 0.343), confirming that piperine markedly enhances autophagosome formation at the ultrastructural level (Figure 6).

**Figure 7:**
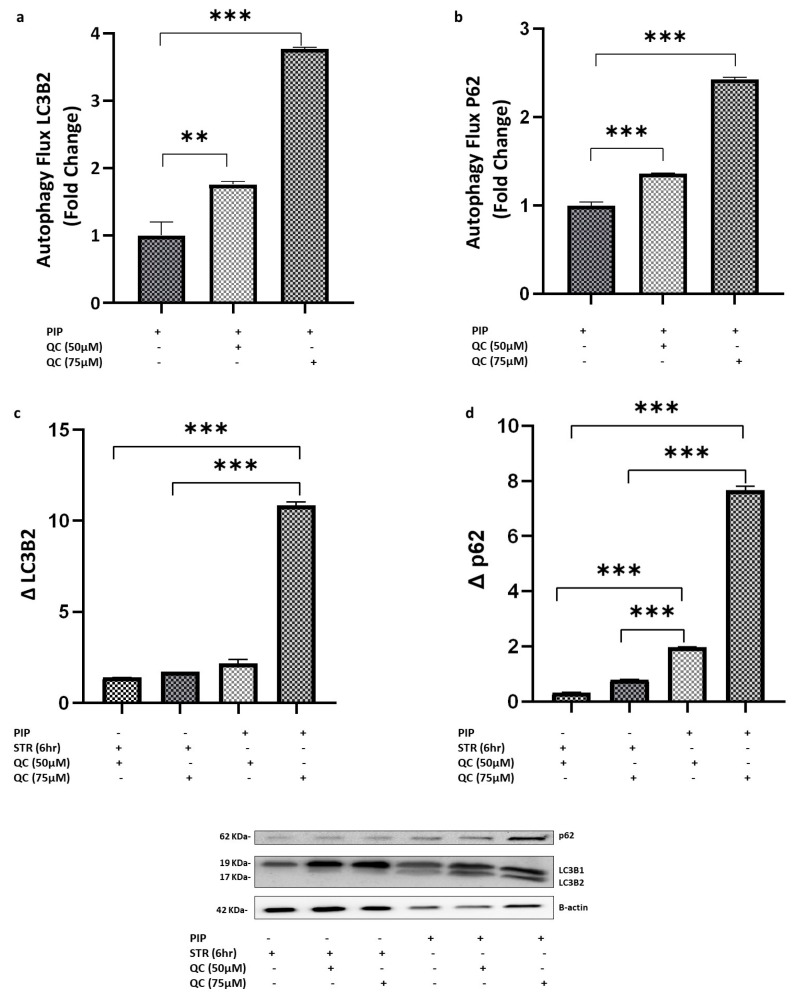
**Piperine Stimulates Autophagic Flux Beyond Starvation Levels in the Presence of Chloroquine.** (**a**,**b**) Co-treatment of piperine-exposed cells with the lysosomal inhibitor chloroquine (QC) increased LC3B-II and p62/SQSTM1 levels, indicating enhanced autophagic flux rather than impaired lysosomal degradation. QC at 50 µM significantly elevated LC3B-II (*p* < 0.01) and p62 (*p* < 0.001), with a further increase observed at 75 µM (*p* < 0.001). (**c**,**d**) Autophagic flux indices (ΔLC3B-II and Δp62) were calculated for starvation and piperine-treated groups. ΔLC3B-II was significantly higher in piperine-treated cells at QC75 compared with starvation (*p* < 0.001), while Δp62 was significantly elevated in piperine-treated cells under both QC50 and QC75 conditions (*p* < 0.001). Data represent mean ± SEM (N = 3). Statistical significance: ** *p* < 0.01, and *** *p* < 0.001 vs. control.

To evaluate autophagic flux, piperine-treated cells were co-treated with chloroquine (QC), a lysosomal degradation inhibitor. Exposure to 50 µM QC resulted in a significant increase in LC3B-II fold change (*p* < 0.01; 95% CI: −3.01 to −1.26) and p62/SQSTM1, a selective autophagy receptor (*p* < 0.001; 95% CI: −2.19 to −1.85), compared with piperine-treated cells alone. These findings indicate an enhancement of autophagic flux rather than lysosomal dysfunction. Notably, increasing the QC concentration to 75 µM produced an even greater elevation in autophagic flux, reflected by higher LC3B-II (*p* < 0.001; 95% CI: −11.69 to −9.95) and p62 (*p* < 0.001; 95% CI: −8.02 to −7.63) levels, as shown in Figure 7a,b.

p62 and LC3B-II accumulation induced by lysosomal inhibition (Δp62 and ΔLC3B-II) were calculated in the starvation and treatment groups using the following formula: For the starvation group:

Δp62 = p62_starvation+QC_ − p62_starvation_

ΔLC3B-II = LC3B-II_starvation+QC_ − LC3B-II_starvation_

For the piperine group (PIP):

Δp62 = p62_PIP_ + QC − p62_PIP_

ΔLC3B-II = LC3B-II_PIP+QC_ − LC3B-II_PIP_

These metrics enable a direct comparison of autophagic flux between starvation and piperine-treated cells. ΔLC3B-II was significantly higher in piperine-treated cells incubated with QC75 compared with the starvation groups (QC50: *p* < 0.001; 95% CI: 8.58 to 10.30; QC75: *p* < 0.001; 95% CI: 8.25 to 10.00), whereas no significant difference was observed with QC50 (*p* > 0.05), as shown in Figure 7c.

For Δp62, piperine-treated cells showed significantly greater QC-induced p62 accumulation compared with starvation under both QC50 (*p* < 0.001; 95% CI: 1.61 to 1.68) and QC75 (*p* < 0.001; 95% CI: 1.14 to 1.21) conditions. Similarly, Δp62 was significantly higher in piperine-treated cells incubated with QC75 compared with the starvation group (QC50: *p* < 0.001; 95% CI: 6.67 to 7.97; QC75: *p* < 0.001; 95% CI: 6.20 to 7.50) as presented in Figure 7d.

#### 2.1.4. NACET Mitigates Piperine-Induced Oxidative Stress and Cytotoxicity

**Figure 8:**
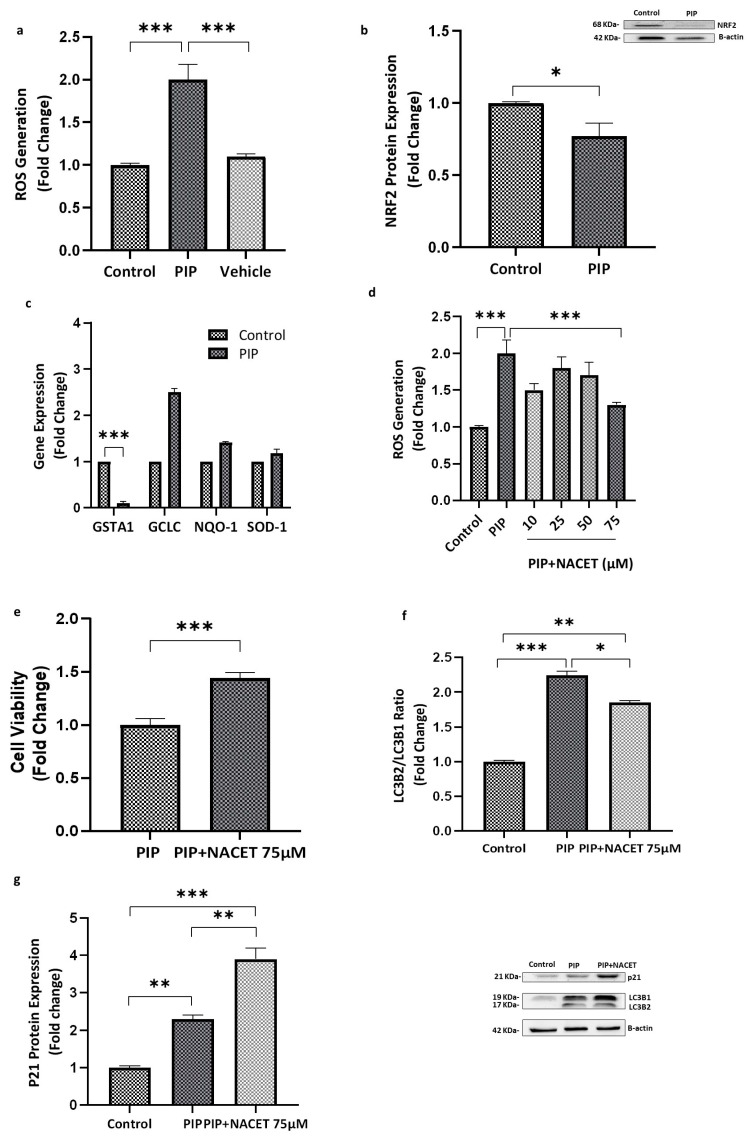
**Piperine Induces Oxidative Stress and Suppresses Antioxidant Responses, While NACET Restores Redox Balance and Cell Viability.** (**a**) Piperine treatment (0.1 mg/mL, 72 h) significantly increased ROS production compared with control and ethanol groups (*p* < 0.001), indicating enhanced oxidative stress. (**b**) Piperine reduced NRF2 expression (*p* < 0.05). (**c**) GSTA1 mRNA levels were markedly downregulated (*p* < 0.001), whereas GCLC, NQO-1, and SOD-1 expression remained unchanged (*p* > 0.05). (**d**) Co-treatment with NACET (75 µM) attenuated piperine-induced oxidative stress (*p* < 0.01). (**e**) NACET (75 µM) significantly improved cell viability in piperine-treated cells (*p* < 0.001). (**f**,**g**) NACET co-treatment also decreased autophagy activation (*p* < 0.05) and increased p21 expression (*p* < 0.05) in piperine-treated cells. Data represent mean ± SEM (N = 3). Statistical significance: * *p* < 0.05, ** *p* < 0.01, and *** *p* < 0.001 vs. control.

Incubation of Caco-2 cells with 0.1 mg/mL piperine for 72 h resulted in a significant elevation in intracellular ROS levels compared with both the control group (*p* < 0.001; 95% CI: 0.35 to 0.97) and the vehicle group (*p* < 0.001; 95% CI: 0.63 to 1.14), indicating pronounced oxidative stress (Figure 8a). This increase in ROS may be attributed, at least in part, to the observed downregulation of NRF2 protein expression in piperine-treated cells relative to the control (*p* < 0.05; 95% CI: 0.11 to 0.34), as shown in Figure 8b.

Analysis of antioxidant-related gene expression further supported this oxidative shift. Piperine treatment significantly reduced GSTA1 mRNA levels (*p* < 0.001; 95% CI: −1.31 to −0.64), whereas no significant changes were detected in GCLC, NQO1, or SOD1 expression (*p* > 0.05) (Figure 8c).

To determine the concentration of NACET capable of mitigating piperine-induced ROS production, cells were pre-incubated with 10, 25, 50, or 75 µM NACET for 2 h prior to piperine exposure. As shown in Figure 8d, only the 75 µM concentration produced a statistically significant reduction in ROS levels (*p* < 0.01; 95% CI: −0.99 to −0.25). Consistent with this antioxidant effect, co-treatment with NACET (75 µM) significantly restored cell viability relative to piperine alone (*p* < 0.001; 95% CI: −0.66 to −0.23), as illustrated in Figure 8e.

Furthermore, NACET co-treatment markedly attenuated piperine-induced autophagy, evidenced by a significant reduction in the LC3B-II/LC3B-I ratio compared with piperine-treated cells (*p* < 0.05; 95% CI: −0.60 to −0.23) (Figure 8f). In contrast, NACET enhanced p21 protein expression relative to piperine alone (*p* < 0.01; 95% CI: 0.31 to 0.52) (Figure 8g). Collectively, these findings demonstrate that oxidative stress is a central mediator of piperine-induced cytotoxicity, and that its attenuation by NACET shifts the cellular response toward improved survival despite persistent cell cycle checkpoint activation.

#### 2.1.5. Piperine Upregulates Occludin Expression and Decreases the Migration Rate of Caco-2 Cells

**Figure 9:**
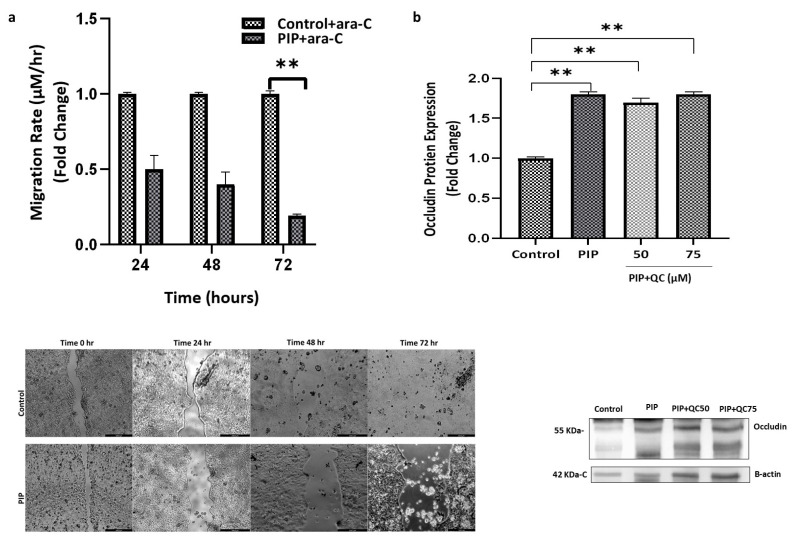
**Piperine reduces migration rate and increases occludin expression in Caco-2 cells.** (**a**) In the presence of the proliferation inhibitor ara-C, piperine significantly reduced the migration rate to 0.0350 µm/h vs. 0.155 µm/h in control + ara-C (*p* < 0.05), indicating an anti-migratory effect independent of cell proliferation. (**b**) Western blot quantification shows significant upregulation of occludin protein after 72 h of piperine treatment compared to control (*p* < 0.01) and ethanol (*p* < 0.05) groups, suggesting enhanced tight junction integrity. Scale bar: 448.6 µM (0 & 24 h) and 224.3 µM (48 & 72 h). Data are presented as mean ± SEM (N = 3). Statistical significance: ** *p* < 0.01 vs. control.

To evaluate the specific anti-migratory effect of piperine independent of its antiproliferative action, cytosine arabinoside (ara-C) was added to both control and piperine-treated groups to inhibit cell proliferation. Comparison of wound closure rates between untreated control cells and control cells treated with ara-C revealed no significant difference in migration (*p* > 0.05), indicating that ara-C at the applied concentration effectively inhibited proliferation without affecting the migratory capacity of Caco-2 cells. In the presence of ara-C, treatment with 0.1 mg/mL piperine resulted in a 0.22-fold change expression relative to control + ara-C cells (*p* < 0.01; 95% CI: 0.33 to 1.21), as shown in Figure 9a.

These findings suggest that piperine suppresses Caco-2 cell migration independently of its antiproliferative effects. This is further supported by the observed upregulation of the tight junction protein occludin in piperine-treated cells (Figure 9b) compared to the control group (*p* < 0.01; 95% CI: 0.34 to 3.48), indicating enhanced cell–cell adhesion associated with reduced migratory capacity. Notably, this increase in occludin expression was not affected by autophagy inhibition, as chloroquine co-treatment did not alter occludin protein levels relative to piperine alone (*p* > 0.05), indicating that occludin regulation in this context is autophagy-independent.

## 3. Discussion

Piperine is a lipophilic amide alkaloid, widely used in traditional medicine for its effectiveness in pain management, allergic diseases, and cardiovascular disorders [22,23,24]. Additionally, numerous in vitro and in vivo studies have demonstrated its potent anti-cancer properties, highlighting its promise as an adjuvant therapy alongside chemotherapy in various cancer types [25,26,27]. In NB4 and MOLT-4 leukemic cell lines, piperine inhibited cell proliferation and induced apoptosis by upregulating Cathepsin-D and proapoptotic genes and proteins, namely Bax and caspase-3 [28]. Furthermore, piperine treatment reduced the viability by activating autophagic cell death in different CRC cells (HCT116 & SW480). This effect was attributed to the inhibition of the AKT/mTOR pathway and oxidative stress activation [29].

The concentration of piperine used in this study (0.1 mg/mL, approximately 350 µM) is substantially higher than the plasma levels achievable in vivo. Pharmacokinetic studies in humans have reported Cmax values ranging from only 3.7 to 595 ng/mL following oral doses of 20–24 mg [29]. Similarly, rodent studies administering 20–54 mg/kg orally have shown Cmax values of 1.1–4.3 µg/mL, again indicating that piperine reaches only low micromolar concentrations even at relatively high doses [30]. These findings highlight the limited bioavailability of piperine and indicate that the concentrations used in our in vitro experiments exceed physiologically attainable levels. We also acknowledge that high concentration of piperine used in this study may elicit non-specific effects typical of lipophilic alkaloids, such as alterations in membrane structure and fluidity, a loss of membrane potential, the disruption of lipid rafts, the inhibition of electron transport chain components, and mitochondrial dysfunction leading to activation of cellular stress pathways. Therefore, the present work should be interpreted primarily as a mechanistic investigation aimed at elucidating cellular pathways modulated by piperine, rather than as a direct prediction of therapeutic efficacy. Future studies employing lower, pharmacologically relevant concentrations or in vivo models will be necessary to evaluate the translational potential of these observations.

In this study, we investigated the inhibitory effect of piperine on colon cancer growth using the Caco-2 cell line. Treatment with piperine significantly decreased the Sub-G0/G1 and G0/G1 populations, did not alter the S-phase fraction, and caused a marked accumulation of cells in G2/M. Together with the absence of nuclear fragmentation, the lack of caspase-3 and PARP cleavage, and the reduction in Sub-G0/G1, these findings indicate that piperine does not induce apoptotic DNA fragmentation but instead triggers a G2/M cell cycle arrest. This effect could be explained by the p53-dependent upregulation of p21 observed in piperine-treated cells. However, because apoptosis was assessed only at the 72 h time point and caspase-independent death pathways were not examined, the possibility of alternative non-apoptotic mechanisms cannot be completely excluded.

The increase in Trypan Blue-positive cells and the decrease in SRB signal further demonstrate that piperine induces cytotoxicity, leading to a loss of membrane integrity and reduced cellular biomass. Overall, piperine disrupts cell cycle progression at the G2/M checkpoint and promotes non-apoptotic cell death, resulting in reduced viability and proliferation.

Autophagy was markedly activated in cells treated with piperine, as evidenced by the prominent accumulation of double-membrane autophagosomes observed under transmission electron microscopy. This was further supported by a significant increase in the LC3B-II/LC3B-I ratio, a widely recognized indicator of autophagosome formation. Although elevated LC3B-II levels can reflect either enhanced autophagy initiation or impaired autophagic flux, our data support the former. Expression levels of lysosomal markers LAMP-1 and cathepsin D were unchanged, indicating preserved lysosomal function. Consistently, lysosome–autophagosome fusion was significantly increased following piperine treatment, as demonstrated by co-localization analysis.

Although chloroquine is commonly used as an autophagy inhibitor, it is not entirely specific to the autophagic pathway. At higher concentrations, CQ can influence additional lysosome-dependent processes, endosomal trafficking, mitochondrial function, and cellular stress responses [31]. These off-target effects may contribute to some of the observed cellular changes. To reduce the likelihood of such non-specific actions, we used concentrations (50–75 µM) lower than those previously reported for Caco-2 cells.

The obtained results showed that chloroquine treatment resulted in a marked accumulation of LC3B-II and p62 proteins compared to cells treated only with piperine, which is consistent with enhanced autophagic flux rather than defective autophagosome degradation. Additionally, the ΔLC3B-II and Δp62 analyses demonstrate that piperine enhances autophagic flux more effectively than starvation, with the strongest differences observed under higher lysosomal inhibition (QC75). This pattern likely reflects the different dynamic ranges and sensitivities of LC3B II and p62 to lysosomal blockade, rather than a strict biological threshold, and consistently supports a higher autophagic flux in piperine-treated cells compared with starvation. This is consistent with previous studies that reported significant activation of autophagy in piperine-treated cells as a response to the stress status [11,32,33].

Additionally, oxidative stress appears to play a role in piperine-mediated growth inhibition. We observed a significant increase in reactive oxygen species (ROS) levels, accompanied by downregulation of *GSTA1*, an antioxidant enzyme involved in detoxifying ROS and protecting cells from oxidative damage. This decrease in the expression of *GSTA1* could be explained by the downregulated transcription factor NRF-2 observed in piperine-treated cells. Thus, the reduction in NRF2 expression likely impaired the antioxidant response, leading to elevated ROS levels. The resulting oxidative stress induced p21 expression and contributed to cell cycle arrest, ultimately suppressing cell proliferation. This pattern aligns with the proposed ROS–NRF2–p21 axis, in which oxidative stress suppresses NRF2 activity and subsequently induces p21.

Importantly, co-treatment with NACET, a ROS scavenger, partially restored Caco-2 viability, supporting the involvement of oxidative stress in the cytotoxic effects of piperine observed in Caco-2 cells. Additionally, the results showed a significant decrease in LC3B2/1 and increase in p21 protein in piperine-treated cells exposed to NACET. Thus, NACET alters the cellular response to piperine by reducing oxidative stress-driven cytotoxicity and shifting cells toward a predominantly cytostatic state, characterized by enhanced p21-dependent checkpoint activation, diminished autophagy, and improved cell viability. These results indicate that inhibition of autophagy by NACET enhances cell viability in piperine-treated cells. This finding suggests that the autophagic response induced by piperine is activated as a result of oxidative stress and it is predominantly cytotoxic rather than cytoprotective. When ROS levels were reduced by NACET, autophagy was suppressed and cell survival improved, implying that piperine induced autophagy contributes to cell death under these conditions.

These observations support previous evidence indicating that piperine increases ROS production [34] and induces cell cycle arrest, reducing the proliferation rate in different colon cancer cells [11,34].

Piperine also inhibited cancer cell migration, a key step in metastasis. This effect was associated with significant upregulation of occludin, a tight junction protein that contributes to cell–cell adhesion and suppresses motility [34]. While the observed correlation suggests a potential role for occludin in mediating piperine’s anti-migratory effect, further mechanistic studies, such as occludin knockdown or overexpression, are needed to confirm its direct involvement. It should also be noted that, although ara-C was used solely as an anti-proliferative control in the migration assay, we did not directly evaluate whether this compound might subtly influence cytoskeletal dynamics or adhesion, and this represents a limitation of the current study. These findings are consistent with reports that occludin expression can increase in response to cellular stress and autophagy activation, potentially enhancing barrier function and reducing motility [35].

## 4. Materials and Methods

### 4.1. Cell Culture

The human colon adenocarcinoma Caco-2 cell line is a widely accepted in vitro model of colorectal cancer due to its origin from human colon carcinoma and its ability to mimic certain features of colon cancer cells [36].

Caco-2 cells at passages 45–50 (American Type Culture Collection, ATCC, Manassas, VA, USA) were used for all in vitro experiments. Cells were cultured in Dulbecco’s Modified Eagle Medium (DMEM) high glucose supplemented with 10% fetal bovine serum, 1% L-glutamine, and 10,000 U/mL penicillin/streptomycin (Euroclone S.p.A., Milan, Italy). Cells were maintained in T75 flasks at 37 °C in a humidified atmosphere containing 5% CO_2_. The medium was changed every 48 h, and cells were passaged upon reaching approximately 80% confluence.

In starvation experiments, Caco-2 cells were seeded as previously described and subjected to nutrient deprivation using low-glucose DMEM lacking glutamine and fetal bovine serum (FBS) for 12 h before the treatment endpoint [37].

In autophagic flux assays, Chloroquine was used as late-stage lysosomal inhibitor [38] and was added at a final concentration of 50 µM or 75 µM for 6 h prior to the treatment endpoint. These concentrations were selected based on previously published work in the same cell line, where CQ was applied at up to 100 µM without inducing excessive cytotoxicity [39]. To minimize potential non-specific cellular effects, cells were treated for 6 h under standard culture conditions.

### 4.2. Piperine Preparation

A stock solution of piperine (10 mg/mL; Sigma-Aldrich, St. Louis, MO, USA) was prepared by dissolving piperine powder in sterilized ethanol. The piperine solution was prepared fresh before each experiment and stored at −20 °C in amber (light-protected) tubes until use to prevent degradation.

Working concentrations of 0.001, 0.01, and 0.1 mg/mL (3.5, 35, and 350 µM, respectively) were prepared by diluting the stock solution in DMEM. An ethanol vehicle (1% *v*/*v*) control was included to account for any effects of the solvent on cell growth. The concentration of 0.1 mg/mL was selected based on preliminary dose–response experiments, representing the IC50 value for piperine cytotoxicity in Caco-2 cells.

### 4.3. Sulforhodamine B (SRB) Assay

The SRB assay was employed to evaluate the cytotoxic effects of piperine on Caco-2 cells and to determine its IC_50_ value, defined as the concentration required to reduce cell viability by 50%, as described previously [40,41]. SRB is an indirect assay for measuring cell proliferation based on total protein content. Caco-2 cells were seeded at 1 × 10^4^ cells/well in 96-well plates and incubated overnight. Cells were then treated with 0.1 mg/mL piperine for 24, 48, and 72 h. After treatment, cells were fixed by adding cold 50% (*v*/*v*) trichloroacetic acid and incubated for 1 h at 4 °C. Plates were washed four times with distilled water and air-dried. A 0.04% (*w*/*v*) SRB solution was added to each well and incubated for 1 h at room temperature. Unbound dye was removed by washing four times with 1% (*v*/*v*) acetic acid. Following drying, the protein-bound dye was solubilized in 50 µL of 10 mM Tris base. Fluorescence was measured using a microplate reader at an excitation wavelength of 488 nm and an emission wavelength of 560 nm.

### 4.4. Cell Cycle Analysis

Cell cycle distribution was assessed by flow cytometry following propidium iodide (PI) staining. Caco 2 cells (5 × 10^5^) were seeded into 6 well plates in complete growth medium containing 10% FBS and allowed to adhere overnight. Cells were then treated with 0.1 mg/mL of piperine or maintained in control medium for 72 h. After treatment, cells were washed three times with PBS, trypsinized, and collected by centrifugation at 0.3× *g* for 5 min. Pellets were fixed in 100% ethanol at −20 °C overnight, washed twice with PBS, and incubated in 0.5 mL PBS containing RNase A (100 µg/mL; R6148, Sigma Aldrich) and propidium iodide (50 µg/mL; P4170, Sigma Aldrich) at 37 °C for 30 min. DNA content was analyzed using a flow cytometer (BD Biosciences, Milan, Italy), and cell cycle profiles were quantified from triplicate samples using FACScan flow cytometer coupled with Cell Quest software v. 3.0 (BD Biosciences, Franklin Lakes, NJ, USA) [42].

### 4.5. Trypan Blue Exclusion Assay

Cell viability was evaluated using the Trypan Blue exclusion assay. Caco-2 cells were seeded at a density of $2\times 10^{5}$ cells per well in a six-well plate and exposed either to piperine (0.1 mg/mL) or to standard culture medium for 72 h. Following treatment, cells were washed with PBS, detached using Trypsin-EDTA, centrifuged, and resuspended in an appropriate buffer to obtain a uniform cell suspension. An equal volume of 0.4% Trypan Blue solution was then added, and the mixture was gently homogenized. After a 3 min incubation period at room temperature, the stained suspension was loaded onto a hemocytometer. Viable cells were identified as unstained, whereas non-viable cells exhibited blue staining. Cell viability was expressed as the percentage of viable cells relative to the total number of cells counted.

### 4.6. Scratch Assay

The scratch assay primarily measures migration, with proliferation acting as a secondary. This assay was performed following the protocol described by Liang et al. (2007) [43]. Caco-2 cells were seeded at 1.5 × 10^5^ cells/well in 24-well plates and incubated until reaching confluence (approximately 24 h).

A linear scratch was made across the cell monolayer using a sterile pipette tip. Images of the wound area were captured immediately (0 h) and at 24, 48, and 72 h post scratch using a LEICA DMI 4000 B phase contrast microscope (Leica Microsystems, Wetzlar, Germany). Wound closure was quantified by measuring the change in the wound area over time. The percentage of wound closure at each time point was calculated using the following formula:

Wound closure (%) = 100 × (Initial area t0 − final area t)/Initial area (t0)

The SRB assay was performed in parallel with the scratch experiment under identical conditions. Wound closure values were subsequently normalized to the corresponding SRB signal to account for differences in cell density and proliferative capacity, as follows:

$$\mathrm{Normali}z\mathrm{ed}\;\mathrm{wound}\;\mathrm{closure}\;(\%)=\frac{\mathrm{Wound}\;\mathrm{closure}\;(\%)}{\mathrm{SRB}\;\mathrm{signal}}$$

To distinguish between migration-dependent and proliferation-dependent effects, two assay conditions were used. For the migration-focused assay, Caco-2 cells were pre-screened with cytosine arabinoside (Ara-C) (Sigma-Aldrich, St. Louis, MO, USA, Cat. No. C1768) to identify a non-toxic dose capable of suppressing proliferation. Based on this screening, 10 µM Ara-C was added to both control and piperine-treated groups 2 h before scratch formation and maintained throughout the assay to minimize proliferation-driven wound closure.

For the proliferation-inclusive assay, Ara-C was not applied, and wound closure therefore reflects the combined contribution of cell migration and cell proliferation.

The formula used to calculate the migration rate is as follows:

Migration rate (µM/h) = Distance at time t − Distance at time 0/time elapse (h)

### 4.7. Western Blot Analysis

Caco-2 cells treated with 0.1 mg/mL piperine for 72 h were lysed in RIPA buffer containing protease and phosphatase inhibitors (Sigma-Aldrich). Cells were scraped, incubated on ice for 15 min, sonicated, and centrifuged at 13,000 *g* for 5 min at 4 °C. Protein concentration was measured using the Bradford assay (Bio-Rad Laboratories, Hercules, CA, USA) [44]. Equal amounts of protein (40 µg) were separated by SDS-PAGE and transferred to nitrocellulose membranes. Membranes were stained with Ponceau S to verify protein transfer, blocked with 5% milk in TBST (0.1% Tween-20) for 1 h, and incubated overnight at 4 °C with primary antibodies against PARP (Cell Signaling Technology, Danvers, MA, USA; Cat. No. 9542S, Lot No. 15; dilution 1:1000), cleaved caspase-3 (Cell Signaling Technology, Danvers, MA, USA; Cat. No. 9661S, Lot No. 43; dilution 1:1000), LC3B (Cell Signaling Technology, Danvers, MA, USA; Cat. No. 43566S, Lot No. 14; dilution 1:1000), cathepsin-D (Cell Signaling Technology, Danvers, MA, USA; Cat. No. 2284S, Lot No. 2; dilution 1:1000), p21 (Cell Signaling Technology, Danvers, MA, USA; Cat. No. 2947S, Lot No. 15; dilution 1:1000), p62 (Cell Signaling Technology, Danvers, MA, USA; Cat. No. 5114S, Lot No. 4; dilution 1:1000), occludin (Cell Signaling Technology, Danvers, MA, USA; Cat. No. 91131S, Lot No. 3; dilution 1:1000), LAMP-1 (Santa Cruz Biotechnology, Dallas, TX, USA; Cat. No. 9091S, Lot No. G1420; dilution 1:1000), and NRF2 (Cell Signaling Technology, Danvers, MA, USA; Cat. No. 12721S, Lot No. 10; dilution 1:1000). Membranes were washed and incubated with appropriate secondary antibodies (Goat anti-Rabbit and Goat anti-Mouse; Bio-Rad; dilution 1:5000) for 1 h at room temperature. Protein bands were visualized using the Clarity Western ECL Substrate kit (Bio-Rad Laboratories, Hercules, CA, USA) and normalized to housekeeping proteins as Beta-Actin expression.

### 4.8. Reactive Oxygen Species (ROS) Assay

ROS production was measured using the H_2_DCFDA probe following the protocol by Neville S Ng et al. (2021) [31]. Caco-2 cells were seeded at 1 × 10^4^ cells/well in 96-well plates and incubated for 24 h before treatment with 0.1 mg/mL piperine for 72 h. After treatment, medium was aspirated, and cells were incubated with 10 µM H_2_DCFDA in PBS for 15 min at 37 °C in the dark. Fluorescence was measured (excitation: 485 nm; emission: 528 nm) using a plate reader (Fluoroskan Ascent fluorimeter, Thermo Labsystems, Helsinki, Finland). Fluorescence data were normalized to cell number using the SRB assay as described above.

To evaluate the impact of oxidative stress on cell growth, N-acetylcysteine ethyl ester (NACET) (Advanced ChemBloks, Hayward, CA, USA; Cat. No. O32426, Lot. No. AC99952A) was applied at different concentrations to reduce ROS levels and protect cells from oxidative damage.

### 4.9. Quantitative Real-Time PCR (qRT-PCR)

Total RNA was extracted from Caco-2 cells, seeded at 7 × 10^5^ cells in T-25 flasks and treated with 0.1 mg/mL piperine for 72 h, using TRIzol Reagent (Invitrogen, Carlsbad, CA, USA). RNA integrity and concentration were assessed with a Nanodrop spectrophotometer (260/280 and 260/230 ratios). cDNA synthesis was performed using the iScript™ cDNA Synthesis Kit (Bio-Rad, Hercules, CA, USA). qRT-PCR was conducted using Applied Biosystems instruments to evaluate the expression of antioxidant genes *NQO-1*, *GSTA1*, *GCLC*, and *SOD1* (Sigma-Aldrich), normalized to *GAPDH*. PCR amplification was conducted on a Bio-Rad thermal cycler using the following program: an initial denaturation at 95 °C for 2 min, followed by 40 cycles of denaturation at 95 °C for 5 s and combined annealing/extension at 60 °C for 30 s. A melt curve analysis was subsequently performed, increasing the temperature from 65 °C to 95 °C in 0.5 °C increments, with a 5 s hold at each step. The heated lid temperature was set at 105 °C to prevent condensation. Relative gene expression was calculated using the 2^−ΔΔCt^ method.

### 4.10. Confocal Fluorescence Co-Localization Analysis

Caco-2 cells were seeded at 200 × 10^3^ cells on glass coverslips placed in 6-well plates and treated as indicated. Cells were fixed with 4% paraformaldehyde and permeabilized using Triton X-100 (0.2% in PBS, Sigma-Aldrich) to allow for antibody access to intracellular structures. After washing with PBS, nonspecific binding was blocked with 1% bovine serum albumin (BSA) for 1 h at room temperature. Cells were then incubated with primary antibodies against LC3B (1:100 in PBS) and LAMP-1 (1:100 in PBS) overnight at 4 °C. Following washing and gentle drying, fluorophore-conjugated secondary antibodies—Alexa Fluor 488 (Invitrogen, Lot No. 1797971) and Alexa Fluor 555 (Invitrogen, Lot No. 2214478)—were applied at a 1:1000 dilution in PBS for 1 h at room temperature. Samples were washed, dried, and mounted using an antifade mounting medium. Confocal images were acquired using sequential scanning to minimize spectral bleed-through, with laser and detector settings optimized to avoid signal saturation. High-magnification images were acquired with a 63× objective. Image analysis was performed using ImageJ/Fiji (version 1.54r (National Institutes of Health, Bethesda, MD, USA)), including background subtraction, thresholding, and quantification of co-localization using Manders’ overlap coefficients within defined regions of interest.

### 4.11. DAPI-Based Fluorescent Nuclear Staining

Caco-2 cells were seeded at 200 × 10^3^ cells on glass coverslips placed in 6-well plates and treated with piperine or normal medium for 72 h. Cells were fixed with 4% paraformaldehyde in PBS for 10–15 min at room temperature and washed three times with PBS. Fixed cells were then incubated with DAPI (4′,6-diamidino-2-phenylindole) at a final concentration of 1 µg/mL in PBS for 5–10 min at room temperature in the dark. After staining, cells were rinsed with PBS to remove excess dye and mounted in an aqueous mounting medium.

Fluorescence images were acquired using a fluorescence microscope (Axio Vision system, Carl Zeiss, Oberkochen, Germany, and LEICA DMRB microscope, Wetzlar, Germany) equipped with a DAPI filter set (excitation ~358 nm, emission ~461 nm). Overview images were obtained with a 10× objective, and high-magnification images were acquired with a 63× objective. Exposure time, gain, and other acquisition parameters were kept constant within each experiment to allow for comparison between samples.

### 4.12. Transmission Electron Microscopy (TEM) Analysis

Caco-2 cells were seeded at 200 × 10^3^ cells in 6-well plates and treated with piperine or normal medium for 72 h. Cells were fixed in 2.5% glutaraldehyde in 0.1 M cacodylate buffer, pH 7.4 (CB) for 2 h, washed in CB, and postfixed in 1.5% potassium ferrocyanide and 1% osmium tetroxide for 1 h, rinse with water and staining in toto in 0.5% uranyl acetate water solution overnight. Samples were dehydrated through a graded ethanol series and embedded in epoxy resin, followed by polymerization at 60 °C. 70 nm ultrathin sections were cut using ultramicrotome Reichert Ultracut E (Wetzlar, Germany) and collected on 100 mesh copper grids. Sections were examined using a transmission electron microscope Tecnai G2 Spirit (FEI Company, Hillsboro, OR, USA) operated at 120 kV, equipped with a CMOS Camera F-216 TVIPS for digital image acquisition.

### 4.13. Data Analysis

The Shapiro–Wilk normality test was used to assess the normal distribution of the obtained data. Student’s *t*-test for independent samples was used to compare the mean of the studied parameters between two independent groups normally distributed. All experiments were performed in triplicates and the results were presented as mean ± standard error of the mean (SEM). GraphPad Prism 8.0.1 and the Statistical Package for the Social Sciences (SPSS) version 20 were utilized for statistical analysis. A *p*-value of less than 0.05 was considered significant. 

## 5. Conclusions

Piperine suppresses Caco-2 cell growth primarily by inhibiting cell proliferation through an oxidative stress-dependent mechanism. In parallel, piperine activates a robust autophagic response while maintaining preserved autophagic flux, as demonstrated by the increased LC3B-II/LC3B-I ratio, intact lysosomal function, enhanced autophagosome–lysosome fusion, and the further accumulation of LC3B-II and p62 following chloroquine treatment. Importantly, co-treatment with the ROS scavenger NACET reduced LC3B-II/LC3B-I levels and significantly improved cell viability, indicating that piperine-induced autophagy is predominantly cytotoxic rather than cytoprotective and is driven by oxidative stress. In addition to its effects on proliferation and autophagy, piperine impaired cancer cell migration and increased expression of the tight junction protein occludin, suggesting a potential role in limiting metastatic behavior. Collectively, these findings highlight the multifaceted anti-cancer actions of piperine—encompassing oxidative stress-mediated growth inhibition, cytotoxic autophagy, and reduced migratory capacity—and support its further investigation as a promising natural compound with therapeutic relevance in colon cancer.

## Data Availability

All data and analysis are available within the manuscript, or upon request to the corresponding authors.

## Abbreviations

The following abbreviations are used in this manuscript:

ACD	Autophagic Cell Death
APC	Adenomatous Polyposis Coli
ATCC	American Type Culture Collection
Bcl-2	B-Cell Lymphoma 2
cDNA	Complementary DNA
CO_2_	Carbon Dioxide
DMSO	Dimethyl Sulfoxide
DMEM	Dulbecco’s Modified Eagle Medium
ECL	Enhanced Chemiluminescence
FBS	Fetal Bovine Serum
GAPDH	Glyceraldehyde 3-Phosphate Dehydrogenase
GCLC	Glutamate-Cysteine Ligase Catalytic Subunit
GSTA1	Glutathione S-Transferase Alpha 1
H2DCFDA	2′,7′-Dichlorodihydrofluorescein Diacetate
IC_50_	Half-Maximal Inhibitory Concentration
KRAS	Kirsten Rat Sarcoma Viral Oncogene Homolog
LC3B	Microtubule-Associated Protein 1 Light Chain 3 Beta
mRNA	Messenger RNA
mTOR	Mammalian Target Of Rapamycin
MTT	3-(4,5-Dimethylthiazol-2-Yl)-2,5- Diphenyltetrazolium Bromide
NQO1	NAD(P)H Quinone Dehydrogenase 1
OD	Optical Density
PARP	Poly (ADP-Ribose) Polymerase
PBS	Phosphate-Buffered Saline
PI3K	Phosphoinositide 3-Kinase
qPCR	Quantitative Polymerase Chain Reaction
RIPA	Radioimmunoprecipitation Assay
RNA	Ribonucleic Acid
ROS	Reactive Oxygen Species
SDS-PAGE	Sodium Dodecyl Sulfate–Polyacrylamide Gel Electrophoresis
SOD1	Superoxide Dismutase 1
SRB	Sulforhodamine B
TBS	Tris-Buffered Saline
TBST	TBS with Tween 20
TCA	Trichloroacetic Acid
WHO	World Health Organization
